# Comparison of left double lumen tube and y-shaped and double-ended bronchial blocker for one lung ventilation in thoracic surgery—a randomised controlled clinical trial

**DOI:** 10.1186/s12871-022-01637-1

**Published:** 2022-04-02

**Authors:** Joachim Risse, Karsten Szeder, Ann-Kristin Schubert, Thomas Wiesmann, Hanns-Christian Dinges, Carsten Feldmann, Hinnerk Wulf, Karl Matteo Meggiolaro

**Affiliations:** 1 Center of Emergency Medicine, University Hospital Essen, Essen, Germany; 2 Department of Anesthesiology and Intensive Care Medicine, Philipps-University, Marburg, Germany; 3 Department of Anesthesiology and Operative Intensive Care Medicine, Diakoneo Diak Hospital Schwäbisch Hall gGmbH, Schwäbisch Hall, Germany

**Keywords:** Y-shaped-bronchial blocker, Left double lumen tube, One lung ventilation, Thoracic surgery

## Abstract

**Background:**

Double lumen tube (DLT) intubation is the most commonly used technique for one lung ventilation. Bronchial blockers (BB) are an alternative, especially for difficult airways. The EZ-bronchial blocker (EZB) is an innovative y-shaped and double-ended device of the BB family.

**Methods:**

A randomised, controlled trial was conducted in 80 patients undergoing elective thoracic surgery using DLT or EZB for one lung ventilation (German Clinical Trial Register DRKS00014816). The objective of the study was to compare the clinical performance of EZB with DLT. Primary endpoint was total time to obtain successful one lung ventilation. Secondary endpoints were time subsections, quality of lung collapse, difficulty of intubation, any complications during the procedure, incidence of objective trauma of the oropharynx and supraglottic space and intubation-related subjective symptoms.

**Results:**

74 patients were included, DLT group (*n* = 38), EZB group (*n* = 36). Median total time to obtain one lung ventilation [IQR] in the DLT group was 234 s [207 to 294] versus 298 s [243 to 369] in the EZB group (*P* = 0.007). Median total time was relevantly influenced by different preparation times. Quality of lung collapse was equal in both groups, DLT group 89.5% were excellent vs. 83.3% in the EZB group (*P* = 0.444). Inadequate lung collapse in five patients of the EZB group resulted in unsuccessful repositioning attempts and secondary DLT placement. Endoscopic examinations revealed significantly more carina trauma (*P* = 0.047) and subglottic haemorrhage (*P* = 0.047) in the DLT group. Postoperative subjective symptoms (sore throat, hoarseness) were more common in the DLT group, as were speech problems.

**Conclusions:**

Using EZB prima facie results in prolonged time to obtain one lung ventilation with equal quality of lung collapse for the thoracic surgeon. If preparation times are omitted in the analysis, the time difference is statistically and clinically not relevant. Our data showed only little evidence for reducing objective airway trauma as well as subjective complaints. In summary both procedures were comparable in terms of times and clinical applicability. Therefore decisions for DLT or EZB should depend more on individual experience, in-house equipment and the individual patient, than on any times that are neither clinically significant nor relevant.

**Trial registration:**

German Clinical Trial Register DRKS00014816, prospectively registered on 07.06.2018

## Background

There are many different airway devices on the market to establish one lung ventilation for thoracic surgery (e.g. double-lumen intubation, bronchus blocker) [1]. The worldwide most used procedure is the double-lumen tube (DLT) technique. DLT intubation has certain disadvantages, including an increased risk of airway trauma [2–4], improper sizing and requirement for replacing it with a single-lumen tube (SLT) if postoperative ventilation is needed in the ICU. DLT intubation is more challenging compared to SLT intubation [2, 5, 6]. These disadvantages have resulted in the development of bronchus blockers (BB). BB such as the Univent torque control blocker, the wire-guided endobronchial Arndt Blocker and the Cohen Flex-tip Blocker represent alternatives to DLT intubation [7–9]. In addition to these established BB devices, the EZ-Blocker™ endobronchial blocker (EZB) (Rüsch, EZ-Blocker, Teleflex, USA), was introduced in 2010 in clinical practice [10]. Contrary to the classic shape of ‘single-ended’ BBs, the ‘double-ended’ EZB has a Y-shaped distal end that mirrors the bifurcation of the trachea and has cuffs on both ends. One lung ventilation is achieved by inflating or deflating the bifurcated cuffs of the left or right side at the relevant main bronchus. The EZB presumably is easy to handle, with a low rate of malposition and fewer dislocations during repositioning and surgical manipulation. Safe and easy use of EZB has been described by researchers before [11, 12]. Previous studies have shown that severe trauma and major complications like bronchial rupture were rare complications when using EZB [13].

To our knowledge, only a few trials have assessed the performance of EZB in comparison with DLT or other BB [14–17]. Recent studies demonstrated longer times for placement of EZB in spite of shorter intubation times with SLT and equal efficiency for SLT plus EZB compared to DLT [16, 17]. Presumably, this EZB device needs longer process times for successful one lung ventilation. In terms of airway trauma and patient-centred outcome parameters, e.g. the incidence of hoarseness, the literature shows conflicting results [16, 17].

We investigated the impact of using SLT plus EZB instead of DLT for one lung ventilation. We focused our investigation on the time needed for correct placement and successful one lung ventilation. In contrast to other studies, in addition to questionnaires; we performed a flexible endoscopic investigation before and 24 h after extubation for greater objectivity.

## Methods

This study was approved by the University's Institutional Review Board (Ethikkommission Marburg, AZ17/18, 16.05.2018) and written informed consent was obtained from all subjects participating in the trial. The trial was registered prior to patient enrolment at the German Clinical Trials registry DRKS (DRKS00014816, Principal investigator: Dr. Joachim Risse, Date of registration: 07.06.2018). This randomised controlled and patient-blinded trial adhered to the CONSORT guidelines. This study was performed in compliance with recognised international standards, including the principles of the Declaration of Helsinki. This study uses established methodology from a previously published work of our Airway Research Group with the focus on thoracic anesthesia; therefore there are similarities and overlaps in the methodology [18]. After providing written informed consent, adult patients scheduled for elective thoracic surgery requiring general anesthesia with the need for one lung ventilation with American Society of Anaesthesiologists physical status I-IV were enrolled from 11.06.2018 until 14.02.2020. Exclusion criteria were patient age < 18 years, non-elective surgery, pregnancy, scheduled rapid sequence induction (RSI), contraindication for DLT insertion or one lung ventilation (risk of aspiration, tumour stenosis, tracheal malformation or obstructions, tumour invasion, small adults, children < 10 years) as well as abnormal physical status of the cervical spine.

### Primary endpoint

Primary endpoint is the total time (s) to obtain successful one lung ventilation for thoracic surgery. This time is defined as time required from the preparation of the device to the correct positioning of the lung isolation device and is a composite measurement of three durations. The total time measured for successful one lung ventilation (s) consists of the following three time segments: preparation time (s), time to successful intubation (DLT or SLT) (s), time for placement of EZB or DLT and one lung ventilation (time for bronchoscopic position check (s) plus the time required for correct placement (s)).

Preparation time (s) consisted of the measured time segments: time for device preparation (s) and time for bronchoscope preparation (s). Preparation time was defined as:

Before intubation, unpacking the devices, assemblage, preparing the required additional materials such as required blocker syringes or clamps (DLT), Balloons EZB and cuff tested (DLT and SLT) and unpacking, assembling and checking the bronchoscopy (for both groups EZB and DLT).

The time for successful intubation was defined as: blade passes mouth opening until positive capnography (visualisation of three expirations by capnography).

The time for bronchoscopic position check (s) was defined as: insertion of the bronchoscope until the current position is recognised. In the event of incorrect position of the device for one lung ventilation, the additional time required for correction was measured. The time for correct placement (s) was defined as: start correction of current position until end of bronchoscopy and approval by the responsible performing anaesthesiologist. All time spans were measured and recorded by an independent investigator.

### Secondary endpoints

In addition to total time to obtain successful one lung ventilation (primary endpoint) we analysed all the different time subsections as secondary endpoints. Further secondary endpoints of this study were quality of lung collapse, number of intubation attempts, malposition, assessment of difficulty, any complications and incidence of intubation-related injuries in both groups.

The definition of malposition was a non-correct device position after intubation with left sided DLT or after intubation with SLT and blind insertion of EZB. The EZB had to rest correctly on the carina after blind insertion and an occlusion of the right and left mainstem bronchus after balloon inflation was certainly possible.

Quality of lung collapse was assessed by the surgeon (blinded to the randomisation result) under direct (thoracotomy) or indirect view (thoracoscopy). Time of the assessment was at least 15 min after one lung was isolated. The lung collapse started at the same time as disinfecting and covering for surgery in the OR. The assessment was made with the first adequate view of the collapsed lung by the surgeon after incision. Classification of lung collapse was made on a three-point-Likert scale as previously described: 1. excellent (complete collapse with perfect surgical exposure); 2. moderate (total collapse, but still some air in the lungs); 3. insufficient (no collapse or partial collapse with interference in surgical procedure)[17, 19].

Regarding Intubation-related injuries we observed only the carina with the bronchoscopic check directly after intubation. Further Intubation-related injuries were investigated by two consecutive flexible endoscopic examinations (at the end of surgery with DLT or ET still in place and on postoperative day one (POD1) without DLT or ET). We examined the oral cavity, the oropharynx, the supraglottic space, the vocal cords and on POD1 also the subglottic space. A follow-up survey by questionnaire according to an established protocol [18] was performed on POD1.

### Sample size calculation

The sample size calculation was based on a previous study [17], which reported a mean placement time of 85 ± 55 s in the DLT group and 192 ± 90 s in the EZB group. Based on these results, an a priori power analysis was performed for the primary endpoint given a beta value of 0.80 and a significance level alpha of 0.05. We calculated a minimum required sample size of 37 patients per group to detect a 15% difference in the time taken for placement of DLT or SLT plus EZB. Because of assumed drop-outs, we added a surcharge of three patients per group to achieve a study sample size of at least 80 patients. Power analysis was performed using G*Power3.1.9.6 for Mac OS X [20, 21].

### Randomisation and allocation concealment

Allocation concealment was achieved using sealed opaque envelopes. Performance blinding was not possible due to study design. Patients and study investigators assessing postoperative outcome parameters were both unaware of the randomisation result. Statistical analysis was performed blinded to study allocation.

### Preoperative assessment

Patients were pre-medicated with 3.75–7.5 mg oral midazolam 45 min before surgery. In the induction area, patients were positioned supine, standard monitoring was applied according to current national guidelines and peripheral intravenous access was established. Patients received pre-oxygenation with 100% oxygen through a mask over five minutes. After pre-oxygenation, anesthesia was induced with 0.3 μg kg^−1^sufentanil and 2 mg kg^−1^ propofol intravenously. Thereafter, 0.6 mg kg^−1^ rocuronium bromide was applied. Neuromuscular monitoring was performed by relaxometry train of four (TOF). Intubation was performed when full relaxation status (TOF 0/4) was reached. Maintenance of general anaesthesia was performed as total intravenous anaesthesia (TIVA) according to the local standards using propofol (4–6 mg kg^−1^ h^−1^) and remifentanil (15–25 µg kg^−1^ h^−1^) adjusted according to the measured anaesthetic depth using bispectral index monitoring (BIS) at a target of 40–60.

**Fig. 1:**
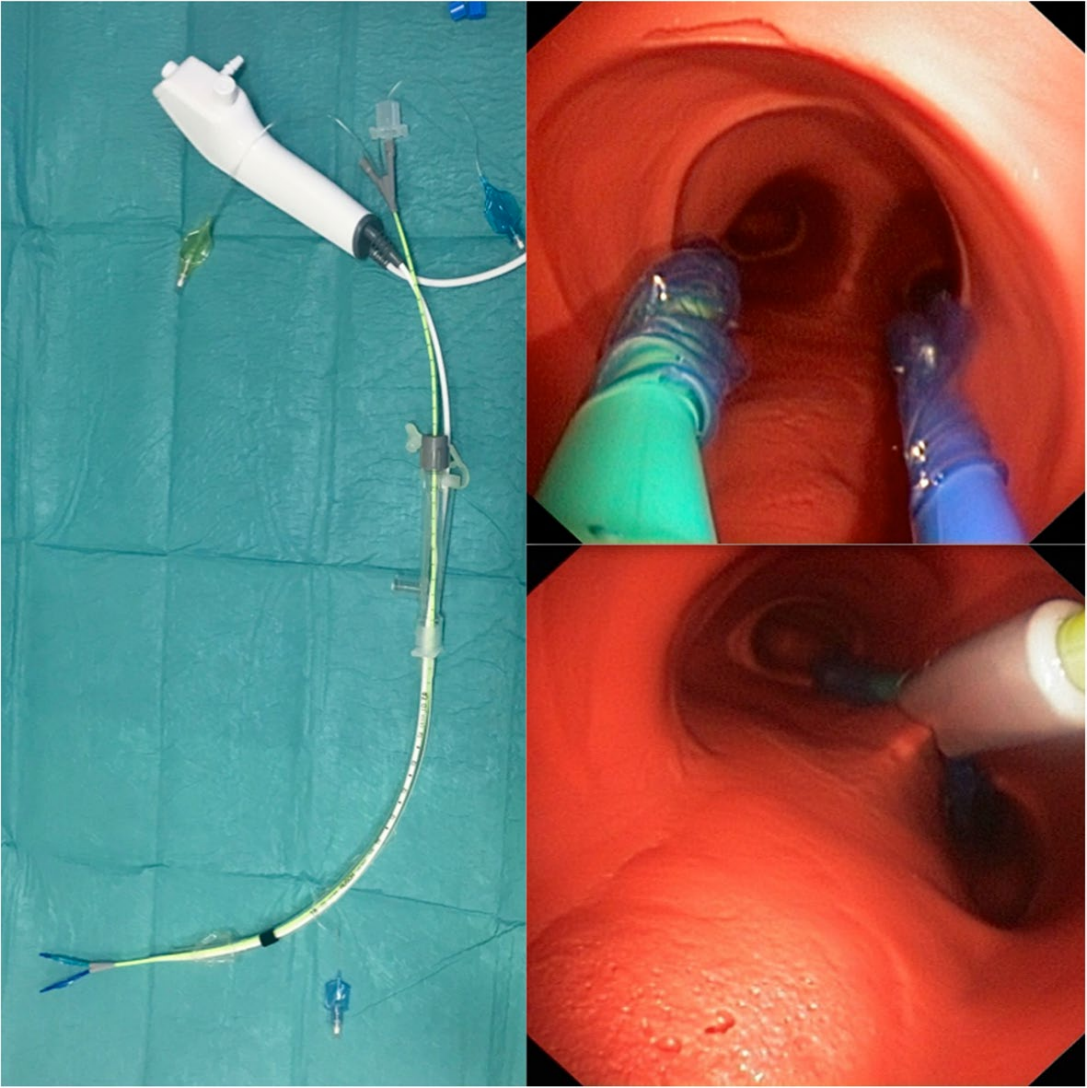
EZ-Blocker and bronchoscope. a. EZ-Blocker (EZB) and Ambu® Broncho aScope 4 slim 3.8/1.2placedthrough a single-lumen tube (SLT) 7.5 mm, b. View with the Ambu® Broncho aScope 4 slim 3.8/1.2on the main carina and c. firm y-shaped position of EZB on the main carina

The size of the DLT (RüschBronchopart; Teleflex Medical GmbH, Dublin, Ireland, 35–41 FR) used was determined for each patient according to Slinger et al. [22]. Only left-sided DLTs were used in this trial. Intubation was performed using a conventional MacIntosh blade (size 3 or 4) as the first line in both groups. In case of difficulties, an intubation attempt with videolaryngoscopy (GVL) was allowed (GlideScope® size 3 or 4). All intubations were performed by the same four experienced anaesthesiologists with extensive training in all types of lung separation techniques including a training course explaining the standardised handling of EZB before starting the study. All bronchoscopies were performed with the Ambu® Broncho aScope 4 slim3.8/1.2 with the associated Ambu® aViewTM monitor (Fig. 1). A bronchoscopic check of the position of DLT or EZB and the time measurement were performed first directly after successful intubation. Correct placement of the respective device was rechecked again after patient positioning before starting the surgical procedure.

### Postoperative assessment

The first endoscopic examination was performed at the end of surgery before extubation orally under general anaesthesia, while the follow-up endoscopic examination was performed transnasal on POD1 under topical anaesthesia. Stored endoscopic video clips were postprocessed for anonymisation and blinding. Thereafter, they were evaluated by three independent investigators (investigator-blinded). The hypopharynx, the vocal cords and the arytenoid cartilage were evaluated on the basis of various criteria. The different criteria were scored from according to the degree of injury (0 = not assessable, 1 = without pathological findings, 2 = minor injuries, 3 = severe injuries). The results (degree of injury 1 to 3) were averaged for further analysis. Second, the patients first completed a questionnaire (Validated H&N35 Quality of Life Questionnaire Head and Neck Module and NRS) to express their subjective symptoms (hoarseness, etc.). NRS scores 1–3 correspond to mild, scores 4–6 to moderate and scores ≥ 7 to severe symptoms. H&N Score ranged from 0–100. A high score correlated with a high degree of complaints and symptoms [23].

### Statistical analysis

Statistical analysis was performed using SPSS (IBM Corp. Released 2016, IBM SPSS Statistics for Windows, Version 25.0, Armonk, NY: IBM Corp.). The normality of the distribution was assessed using the Shapiro–Wilk test. All values for descriptive statistics and outcome parameters were non-normally distributed. All non-normally distributed data are presented as median and interquartile range (IQR). Dichotomous outcome parameters are expressed as events (percentages). Non-parametric data were analysed using the Mann–Whitney U-test. *P* < 0.05 was considered statistically significant.

## Results

**Fig. 2:**
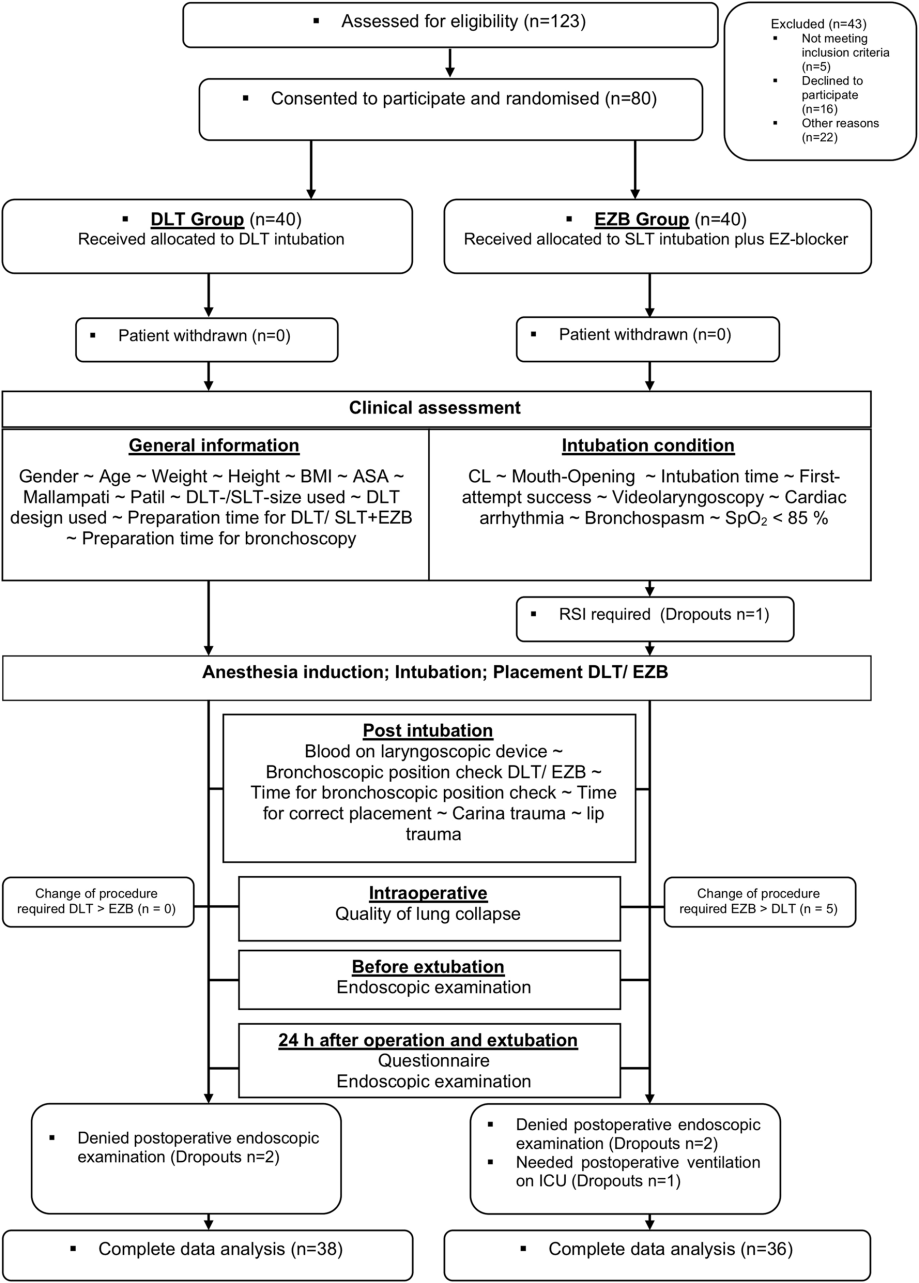
CONSORT and study flow diagram

For the study 123 patients were assessed for eligibility, in the period from 11.06.2018 until 14.02. 2020. Out of these patients 43 were excluded (Not met inclusion criteria *n* = 5, declined to participate *n* = 16, other reasons *n* = 22). After the exclusion 80 patients were randomised for our study. Finally 74 completed the study and were included in the final analysis (Fig. 2). Two patients in the DLT group and four patients in the EZB group were excluded from the final analysis. Two participants randomised to the DLT group and two in the EZB group refused postoperative nasal endoscopic examination. In the EZB group, one participant needed rapid sequence induction and one participant needed long-term postoperative ventilation and was lost to follow-up. All six participants were excluded from the final analysis due to relevant study protocol violation, as predefined (Fig. 2).The groups showed no significant differences in demographics, preoperative airway assessments and descriptive intubation data (Table 1).

**Table 1:** Biometric data and descriptive intubation data of patients enrolled in the study. Data are presented as median [IQR] or numbers (percentage)

Parameter	DLT (*n *= 38)	EZB (*n* = 36)
**Gender (male/female)**	25/13	23/13
**Age (years)**	64 [55 to 74]	64 [59 to 72]
**Weight (kg)**	76 [63 to 90]	81 [70 to 101]
**Height (cm)**	174 [168 to 178]	174 [165 to 181]
**Body mass index (kg m**^**−2**^**)**	25.04 [21.34 to 28.72]	26.42 [24.34 to 30.81]
**ASA** *n* (%)**:**		
I	0 (0%)	1 (3%)
II	9 (24%)	6 (17%)
III	26 (68%)	27 (75%)
IV	3 (8%)	2 (5%)
**Mallampati score** *n* (%)**:**		
I	13 (34%)	14 (39%)
II	20 (53%)	19 (53%)
III	5 (13%)	3 (8%)
IV	0 (0%)	0 (0%)
**Cormack-Lehane score** *n* (%)**:**		
I°	22 (58%)	20 (55%)
II°	13 (34%)	11 (31%)
III°	3 (8%)	5 (14%)
IV°	0 (0%)	0 (0%)
**Patil-Test (cm):**	8.0 [7 to 9]	8.3 [7.5 to 9]
**Mouth opening relaxed (cm):**	3.5 [3 to 4.5]	3.5 [3.5 to 4]

### Primary endpoint

**Fig. 3:**
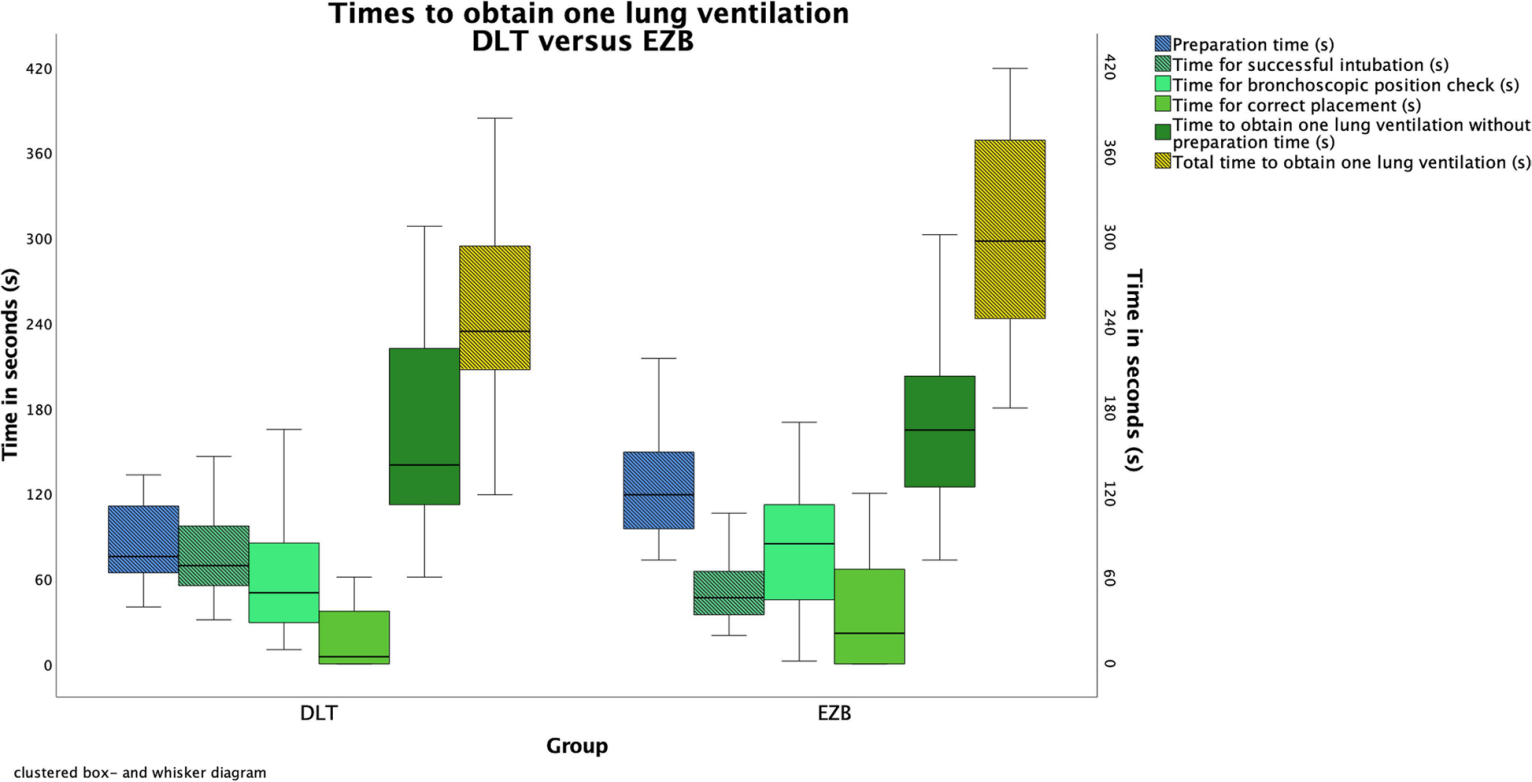
Clustered box-and-whisker diagram of process times for lung separation

Median total time until successful one lung ventilation in the DLT group was significantly shorter in the EZB group compared to DLT group 234 s [207 to 294] versus 298 s [243 to 369](*P* = 0.007). The time difference is only due to the different preparation times for the devices (see Table 2 and Fig. 3). If preparation time is omitted from analysis the groups did not longer differ statistically. Time for successful one lung ventilation without preparation time was 140 s [112 to 222] in the DLT group and 165 s [125 to 203] (*P* = 0.479).

**Table 2:** One lung ventilation data: Time for intubation, preparation of the devices, bronchoscopic control. Data are presented as median [IQR] or numbers (percentage)

Parameter	DLT (*n* = 38)	EZB (*n* = 36)	Mann Whitney U-test (*P*-Value)
**Primary endpoint (Total time)**			
**Total time for one lung ventilation (s) (preparation/intubation/isolation)**	234 [207 to 294]	298 [243 to 369]	0.007*
**Secondary endpoints (Time subsections)**			
**Preparation time (s)** **(device + bronchoscope)**	76 [64 to 111]	119 [95 to 149]	< 0.001*
**Time for successful intubation (s)**	69 [55 to 97]	47 [35 to 65]	< 0.001*
**Time for bronchoscopic position check (s)**	50 [29 to 94]	85 [45 to 113]	0,107
**Time for correct placement (s)**	5 [0 to 38]	22 [0 to 68]	0,051
**Time for bronchoscope preparation (s)**	28 [19 to 35]	23 [20 to 32]	0.349
**Time for device preparation (s)**	49 [41 to 70]	97 [74 to 117]	< 0.001*
**Time for one lung ventilation without preparation time (s)**	140 [112 to 222]	165 [125 to 203]	0.479
**Correct device position after intubation** *n* (%)**:**			
yes	27 (71%)	16 (44%)	0.021*
no	11 (29%)	20 (56%)	
**Method of intubation** *n* (%)**:**			
DL	29 (87%)	28 (86%)	0.927
GVL	9 (13%)	8 (14%)	
**Change method of intubation** *n* (%)**:**			
yes	3 (8%)	2 (6%)	0.691
no	35 (92%)	34 (94%)	
**First attempt success** *n* (%)**:**			
yes	29 (76%)	32 (89%)	0.158
no	9 (24%)	4 (11%)	
**Intubation attempts** *n* (%)**:**			
1	27 (71%)	29 (81%)	0.32
2	10 (26%)	7 (19%)	
3	0 (0%)	0 (0%)	
> 3	1 (3%)	0 (0%)	

^*^Statistically significant

### Secondary endpoints

Regarding the secondary endpoints, the preparation time was significantly longer in the EZB group with 119 s [95 to 149] compared to the DLT group 76 s [64 to 111](*P* = 0.001). The time to successful intubation in the EZB group was significantly shorter with 47 s [35 to 65] versus 69 s [55 to 97] in the DLT group (*P* = 0.001). The times for correct one lung ventilation after intubation did not differ between the two groups (Table 2).

First-attempt success did not differ significantly between the DLT group (76%) and the EZB group (89%) (*P* > 0.05) (Table 2). There was no statistically significant difference between groups regarding the frequency of intubation attempts (*P* > 0.05).

During bronchoscopic control, correct positioning of the DLT or SLT plus EZB for selective lung ventilation was reported in 71% in the DLT group directly after successful endobronchial intubation, whereas only 44% of the devices in the EZB group were adequately positioned (*P* = 0.021) (Table 2).

**Fig. 4:**
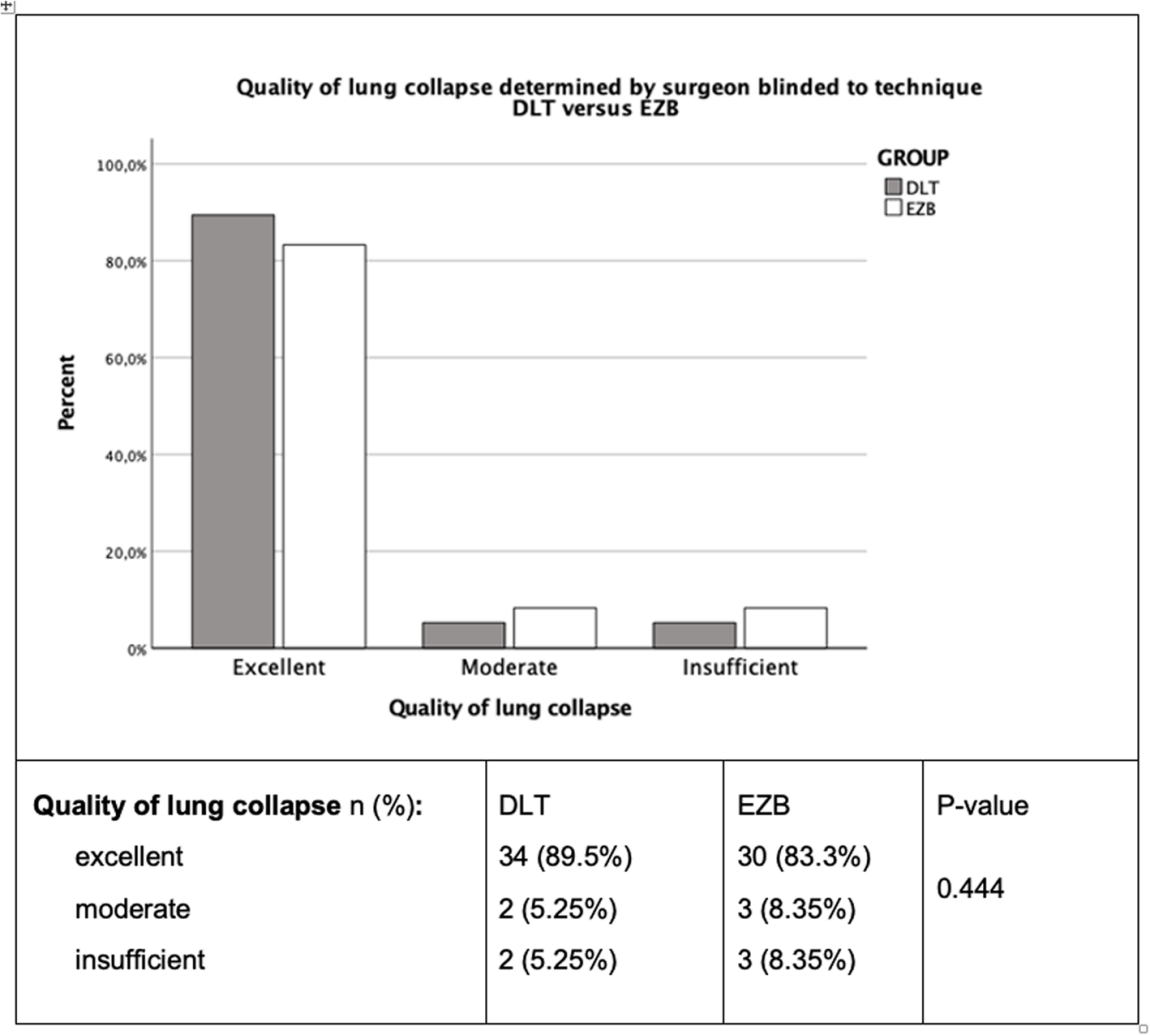
Bar chart and results of the quality of lung collapse

The quality of lung collapse was equal in both groups (DLT group 89.5% were excellent vs. 83.3% EZB Group (*P* = 0.444)) (Fig. 4). A repositioning attempt was reported in 10.5% (4/38) in the DLT group and in 16.6% (6/36) in the EZB group. Inadequate lung collapse in five patients of the EZB group resulted in unsuccessful repositioning attempts and secondary DLT placement. These five crossover cases showed adequate one lung ventilation after DLT placement. With adequate lung collapse and correct positioning of DLT or EZB, no secondary dislocation occurred intraoperatively in any group.

There were significantly more carina traumas in the DLT group (*P* = 0.047) (Table 3). There was no other significant difference in terms of direct complications after intubation between the two groups.

**Table 3:** Assessment of difficulty and complications. Data are presented as number (percentage)

Parameter	DLT (*n *= 38)	EZB (*n* = 36)	Mann Whitney U-test (*P-*value)
**SpO**_**2**_** < 85%** *n* (%)**:**			
yes	1 (3%)	0 (0%)	0.33
no	37 (97%)	36 (100%)	
**Bronchospasm** *n* (%)**:**			
yes	1 (3%)	0 (0%)	0.33
no	37 (97%)	36 (100%)	
**Cardiac arrhythmia** *n* (%)**:**			
yes	1 (3%)	0 (0%)	0.33
no	37 (97%)	36 (100%)	
**Blood on device** *n* (%)**:**			
yes	3 (8%)	0 (0%)	0.087
no	35 (92%)	36 (100%)	
**Carina trauma** *n* (%)**:**			
yes	4 (11%)	0 (0%)	0.047*
no	34 (89%)	36 (100%)	

^*^Statistically significant

When analysing the postoperative questionnaires (H&N35 and NRS scores) to record the subjective symptoms after intubation, significantly more incidence of sore throat, hoarseness and speech problems were found in the DLT group. With the NRS score, the items sore throat (*P* = 0.009) and hoarseness (*P* = 0.02) were significantly lower in the EZB group (Table 4). With the H&N35 score the two items sore throat (*P* = 0.015) and speech problems (*P* = 0.047) were significantly lower in the EZB group (Table 5). However, there was no difference in the valid total H&N35 score between the two groups (*P* = 0.064).

**Table 4:** Results of parameters additionally examined with numerical rating scale (NRS). NRS scores 1–3 correspond to mild, scores 4–6 to moderate and scores ≥ 7 to severe symptoms. Values are expressed as the number of patients or as the total number in percent

Parameter of the NRS score	DLT (*n* = 38)	EZB (*n* = 36)	U-test (*P*-value)
**Sore throat** n (mild/moderate/severe) (total in %)	9/0/0 (23.7%)	1/0/0 (2.8%)	0.009*
**Dysphagia** n (mild/moderate/severe) (total in %)	6/1/0 (18.4%)	1/2/0 (8.3%)	0.247
**Cough** n (mild/moderate/severe) (total in %)	11/9/0 (52.6%)	17/5/0 (61.1%)	0.916
**Hoarseness** n (mild/moderate/severe) (total in %)	6/9/3 (47.4%)	9/1/0 (27.8%)	0.02*

^*^Statistically significant

**Table 5:** Results of relevant selected parameters from evaluation of the H&N35 Quality of Life Questionnaire Head and Neck Module (H&N Score). Data are presented as median [IQR]

Parameter (H&N Score)	DLT (*n* = 38)	EZB (*n* = 36)	Mann Whitney U-test (*P*-value)
Sore throat	0 [0 to 33]	0 [0 to 0]	0.015*
Dysphagia	0 [0 to 0]	0 [0 to 0]	0.202
Cough	33 [0 to 33]	33 [0 to 67]	0.411
Hoarseness	0 [0 to 67]	0 [0 to 33]	0.077
Dry mouth	67 [0 to 67]	33 [0 to 67]	0.429
Viscous mucus	33 [0 to 33]	0 [0 to 33]	0.131
Paraesthesia	0 [0 to 0]	0 [0 to 0]	0.143
Speech problems	0 [0 to 33]	0 [0 to 0]	0.047*
Mouth opening problems	0 [0 to 0]	0 [0 to 0]	0.071
Toothache	0 [0 to 0]	0 [0 to 0]	0.613
Total H&N35 score	17 [7 to 23]	12 [7 to 17]	0.064

^*^Statistically significant

In contrast to the subjective symptoms, postoperative endoscopic examinations revealed significant differences in the EZB group compared to the DLT group in terms of objective trauma, i.e. subglottic haemorrhage (*P* = 0.047) (Table 6).

**Table 6:** Data of reported intubation related injuries from two transnasal endoscopic examinations; before and 24 h after extubation. All different criteria were scored from 0 to 3. (0 = not assessable, 1 = without pathological findings, 2 = minor injuries, 3 = severe injuries).Values are expressed as median (5^th^-25^th^-75^th^-95^th^ percentile)

Parameter	DLT pre-extubation		EZB pre-extubation	U-test (*P*-value)	DLT 24 h post-extubation	EZB 24 h post-extubation	U-test (*P*-value)	
Vocal cord swelling		1.00 (1.00–1.00–1.00–1.67)	1.00 (1.00–1.00–1.00–2.00)	0.457	1.00 (1.00–1.00–1.33–1.67)	1.00 (1.00–1.00–1.33–1.67)		0.711
Vocal cord redness		1.00 (1.00–1.00–1.00–1.33)	1.00 (1.00–1.00–1.00–1.33)	0.659	1.00 (1.00–1.00–1.00–1.33)	1.00 (1.00–1.00–1.00–1.50)		0.369
Vocal cord oedema		1.00 (1.00–1.00–1.00–1.33)	1.00 (1.00–1.00–1.00–1.67)	0.111	1.00 (1.00–1.00–1.00–1.33)	1.00 (1.00–1.00–1.00–1.50)		0.974
Vocal cord erythema		1.00 (1.00–1.00–1.00–1.00)	1.00 (1.00–1.00–1.00–1.00)	1.0	1.00 (1.00–1.00–1.00–1.33)	1.00 (1.00–1.00–1.00–1.50)		0.795
Vocal cord hematoma		1.00 (1.00–1.00–1.00–1.00)	1.00 (1.00–1.00–1.00–1.33)	0.539	1.00 (1.00–1.00–1.00–1.67)	1.00 (1.00–1.00–1.00–1.67)		0.543
Vocal cord haemorrhage		1.00 (1.00–1.00–1.00–1.00)	1.00 (1.00–1.00–1.00–1.00)	0.325	1.00 (1.00–1.00–1.00–1.33)	1.00 (1.00–1.00–1.00–1.33)		0.207
Vocal cord granuloma		1.00 (1.00–1.00–1.00–1.00)	1.00 (1.00–1.00–1.00–1.00)	0.325	1.00 (1.00–1.00–1.00–1.33)	1.00 (1.00–1.00–1.00–1.00)		0.179
Vocal cord mobility		–	–	–	1.00 (1.00–1.00–1.00–1.33)	1.00 (1.00–1.00–1.00–1.33)		0.874
Arytenoid cartilage trauma		1.00 (1.00–1.00–1.00–1.00)	1.00 (1.00–1.00–1.00–1.00)	0.332	1.00 (1.00–1.00–1.00–1.33)	1.00 (1.00–1.00–1.00–1.33)		0.564
Hypopharynx redness		1.33 (1.00–1.00–1.50–2.00)	1.33 (1.00–1.00–1.33–1.67)	0.995	1.00 (1.00–1.00–1.33–1.33)	1.00 (1.00–1.00–1.33–1.67)		0.930
Hypopharynx oedema		1.00 (1.00–1.00–1.00–1.67)	1.00 (1.00–1.00–1.33–1.67)	0.065	1.00 (1.00–1.00–1.00–1.33)	1.00 (1.00–1.00–1.00–1.33)		0.364
Hypopharynx hematoma		1.00 (1.00–1.00–1.00–1.33)	1.00 (1.00–1.00–1.00–1.50)	0.522	1.00 (1.00–1.00–1.00–1.33)	1.00 (1.00–1.00–1.00–1.33)		0.910
Hypopharynx haemorrhage		1.00 (1.00–1.00–1.33–2.00)	1.00 (1.00–1.00–1.33–2.00)	0.298	1.00 (1.00–1.00–1.00–1.33)	1.00 (1.00–1.00–1.00–1.33)		0.564
Subglottic redness		–	–	–	1.00 (1.00–1.00–1.00–1.67)	1.00 (1.00–1.00–1.00–1.00)		0.266
Subglottic oedema		–	–	–	1.00 (1.00–1.00–1.00–1.00)	1.00 (1.00–1.00–1.00–1.00)		0.332
Subglottic hematoma		–	–	–	1.00 (1.00–1.00–1.00–1.50)	1.00 (1.00–1.00–1.00–1.00)		0.061
Subglottic haemorrhage		–	–	–	1.00 (1.00–1.00–1.33–1.67)	1.00 (1.00–1.00–1.00–1.00)		**0.047**

^*^Statistically significant

## Discussion

Prima facie our study showed a significantly prolonged time required to obtain successful one lung ventilation using EZB, but notably only influenced by the prolonged preparation time in contrast to DLT. In addition, there was a significantly higher incidence of malpositioned devices in the EZB group after blind insertion, but on the other hand we found a significantly higher rate of airway trauma as well as subjective complaints in the DLT group.

### Process times

Prolonged intubation times for EZB were shown in previous studies and have a greater risk of hypoxia [16, 17]. However, the combination of EZB with SLT guarantees a secured airway and oxygenation while the EZB is positioned.

The prolonged total time for successful one lung ventilation was notably influenced by the prolonged preparation time and preparation time is not clinically relevant. Preparations are done in advance of preparing patients for surgery. Preparation time therefore has no influence on airway injuries or other complications such as desaturation. A shorter intubation time for SLT seems to be relativised based on the time required to obtain successful one lung ventilation. This seems logical, because SLT plus EZB is a two-step procedure. A shorter intubation time is certainly relevant in patients with a higher risk of aspiration and desaturation.

In our opinion, the marginal difference in the process times is in the end not clinically relevant. In the case of emergency intubation due to pulmonary bleeding, rapid intubation with SLT plus EZB may offer advantages, because DLT intubation requires more expertise. Therefore, when using EZB, there should also be a focus on non-elective use in the intensive care units and emergency rooms.

### Incidence of device malposition

Previous studies of Ruetzler et al. showed a lower incidence of malpositions with DLT (10%) for blind insertion without flexible fibre optic bronchoscopy (FOB) compared to EZB (79%) [17]. In contrast, Mourisse and colleagues showed a very high incidence of initial malpositions and need for repositioning the device during FOB in both groups (85% DLT vs. 74% EZB) [16]. Contrary to Ruetzler et al. and us the malposition was methodically defined differently. With 29% initial malpositions, we had a significantly lower incidence for DLT, similar to the results by Ruetzler et al. With EZB the position during FOB had to be corrected in more than half of the cases (56%). Initial malpositions are caused by too-deep positioning of the SLT used for introducing the EZB [11]. This was the most common cause for malposition in our study cohort as well. Because of the high incidence of malposition we underline the recommendation to use FOB for the placement of EZB [16]. A correspondingly thin bronchoscope and an endotracheal tube as large as possible are obligate in order to fit parallel with EZB.

### Success and quality of lung collapse

Overall, we had good results for both techniques based on successful OLV. In all intubations with SLT, we did not have a single case of entrapment of an EZB in the Murphy eye [24].

OLV with EZB was described before as a safe and easy technique with good quality of lung collapse [11, 12]. We were able to confirm these findings with our data. The only two randomised trials between DLT and EZB so far have shown no difference in quality of lung collapse assessed by the surgeon [16, 17].

Prima facie, our study results seem to confirm the previous ones. However, there was only a change in method to a left-sided DLT in five of six cases in the EZB group, if the quality of collapse was not excellent. Conversely, in the DLT group with repositioning of the left-sided DLT, sufficient surgery conditions intraoperatively could be achieved in all cases. In all five cases, the reason was the inadequate closing of the aperture of the right upper lobe bronchus. Adequate OLV by EZB seems to cause difficulties, especially for right-sided thoracotomies [12, 25]. The shorter distance between the main carina and the aperture of the right upper lobe can make the use of EZB difficult, especially in case of a tracheal bronchus. In our cases ameliorating the position of the EZB by retracting the EZB under bronchoscopic view until the balloon is situated at the aperture of the right upper lobe was frustrated. A simple reason for these five method changes could be a lack of experience in handling the EZB, because EZB was the newest BB that we used in the clinical routine in our department.

### Incidence of airway trauma

With equal quality of lung collapse and less traumatic damage of the glottis and subglottic level, the extended time to separate the lungs with EZB takes a back seat. In our study, more carina trauma and subglottic haemorrhage in the endoscopic follow-up were the objective evidence for airway trauma. Severe trauma such as bronchus perforation did not occur in our patients [13] and no patients required intervention to treat an airway damage.

Our findings are in agreement with the findings of Mourisse et al., who described placing EZB took more time and had a lower rate of airway injury [16]. In contrast we found significantly more carina trauma by first endoscopic follow-up in the DLT group. We expected more carina trauma with EZB, because of the initial blind insertion and the y-shaped distal end sticking fixed on the carina. We conclude that pressure and forces during blind introduction and rotation manoeuvre of the left-sided DLT in the tracheal part might cause more airway trauma.

Regarding subjective symptoms, there are controversial results in the literature by Mourisse et al. and Ruetzler et al. [16, 17]. Our results show a significant trend to increased subjective complaints after DLT. We could show for individual items of H&N35 score and items of NRS score significantly more subjective symptoms 24 h after extubation in the DLT group. But the recognised total score of H&N35 questionnaire showed no significant difference. The question remains as to whether the questionnaires used are sensitive enough to record differences in subjective symptoms.

According to our results and experience with the EZB, we would not deviate anymore from the general recommendation for all BB’s, including the EZB, to always use bronchoscopy for placement from the beginning of the insertion. This might ultimately shorten the procedure and prevent even more airway trauma of the carina.

### Limitations

Although time to intubation or time to successful one lung ventilation is commonly used in airway studies, because they are methodologically easy to compare, the clinical relevance remains questionable. In our case it is important to note that preparation time has not necessarily been incorporated in the intubation procedure and time measurement to obtain successful one lung ventilation, because due to the different preparation times, there is no increased risk for patients such as desaturation or other complications. Operators were not blinded to the intubation device used. Nevertheless, the patient and follow-up endoscopic examinations were anonymised and blinded. Further limitation might be that the operators were not equally experienced with both devices. A small number of patients with an expected difficult airway (Mallampati 3 and 4) and the low incidence of predicted difficult airways (CL 3 and 4) is a further limitation of our study. EZB is an important technique for OLV in patients with an expected difficult airway. Furthermore, we cannot exclude injuries by the endoscopic flexible bronchoscopies at the follow-up itself, although they were all done by experienced investigators. Lastly, we did not investigate a long-term outcome with our follow-up at 24 h after surgery.

## Conclusions

Prima facie our data showed prolonged total time to obtain one lung ventilation in the EZB group with equal quality of lung collapse for the thoracic surgeon. The prolonged total time for successful one lung ventilation in the EZB group was notably influenced by the prolonged preparation time for SLT plus EZB. If preparation times are omitted in the analysis the time difference to obtain successful one lung ventilation is statistically and clinically not relevant.

In addition to no real time difference in airway management to obtain successful one lung ventilation, there was no subjective difference for the patient as a relevant patient-centred outcome parameter, despite some advantages due to less objective airway injuries.

In summary, our randomised controlled trial showed that both procedures were comparable in terms of times to obtain one lung ventilation and clinical applicability. Therefore decisions for DLT or EZB should depend more on individual experience, in-house equipment and the individual patient, than on any times that are neither clinically significant nor relevant.

Further studies are needed to underline the advantages of EZB for one lung ventilation in thoracic surgery.

## Authors' information

Our study adheres to CONSORT guidelines and we included in the section supporting information a completed CONSORT checklist and CONSORT diagram

## Data Availability

The data that support the findings of this study are available from the corresponding author. The datasets used and analysed during the current study are available from the corresponding author on reasonable request.

## Abbreviations

BB: Bronchial blocker
BIS: Bispectral index monitoring
CL: Cormack and Lehane
DL: Direct laryngoscopy
DLT: Double lumen tube
EZB: EZ-bronchial blocker
FOB: Flexible fibreoptic bronchoscopy
GVL: GlideScope videolaryngoscopy
ICU: Intensive care unit
IQR: Interquartile range
NRS: Numeric rating scale
OR: Operating room
OLV: One-lung-ventilation
POD1: Postoperative day one
RSI: Rapid sequence induction
SLT: Single lumen tube
SOP: Standard operating procedure
TIVA: Total intravenous anaesthesia
TOF: Train of four
VATS: Video-assisted thoracoscopic surgery
